# Increased trend in extracorporeal membrane oxygenation use by adults in the United States since 2007

**DOI:** 10.1186/s13104-015-1678-7

**Published:** 2015-11-18

**Authors:** Alicia K. Gerke, Fan Tang, Joseph E. Cavanaugh, Kevin C. Doerschug, Philip M. Polgreen

**Affiliations:** Department of Internal Medicine, University of Iowa, 200 Hawkins Dr., Iowa, IA 52242 USA; Department of Biostatistics, University of Iowa, 105 River Street, Iowa, IA 52242 USA

**Keywords:** Extracorporeal membrane oxygenation, Trends, Outcome assessment, Mortality, Hospitalization, Epidemiology

## Abstract

**Background:**

Extracorporeal membrane oxygenation (ECMO) has been increasingly studied as a life support modality, but it is unclear if its use has changed over time. Recent publication shows no significant trend in use of ECMO over time; however, this report does not include more recent data. We performed trend analysis to determine if and when the use of ECMO changed in the past decade.

**Results:**

We identified hospitalizations (2000–2011) in the Nationwide Inpatient Sample during which ECMO was recorded. We used a segmented linear regression model to determine trend and to identify a temporal change point when rate of ECMO use increased. ECMO use gradually grew until 2007, at which time there was a dramatic increase in the rate (p = 0.0003). There was no difference in mortality after 2007 (p = 0.3374), but there was longer length of stay (p = 0.0001) and smaller percentage of women (p = 0.005).

**Conclusions:**

There has been a marked increase in ECMO use since 2007. As ECMO use becomes more common, further study regarding indications, cost-effectiveness, and outcomes is warranted to guide optimal use.

## Findings

### Background

Extracorporeal membrane oxygenation (ECMO) provides temporary means of support for cardiopulmonary failure when conventional methods fail. First used in adults during the 1970s, a renewed interest has occurred due technological advances, increased safety, and decreased complications [1]. In addition, renewed interest in ECMO by intensivists has also been due to a large randomized controlled trial suggesting efficacy of ECMO in respiratory failure, reports of successful use in respiratory failure cases during the H1N1 influenza pandemic, and in more recent reports as a bridge to lung transplantation [2–5].

A recent study has suggested that the trend in ECMO admissions is not significantly increasing, although these data are reported only up to 2009 [6]. However, it does appear that hospital charges and length of stay are increasing, perhaps associated with a shift in use of ECMO to patients with worse outcomes [6]. Thus, the objective of this study was to determine how the national frequency of ECMO use has changed over the past decade in the United States (US) with a larger number of years of data than previously reported, and to determine the point in time point at which the use may have changed. We also describe the demographics, length of stay, and mortality of patients receiving ECMO during hospitalization. Understanding trends will help hospitals plan for increases in use and help inform future research studies regarding safety, efficacy, and appropriate indications of use.

## Methods

All data were extracted from the Nationwide Inpatient Sample, the largest all-payer database of national discharges in the US [7]. The database is maintained as part of the Healthcare Cost and Utilization Project by the Agency for Healthcare Research and Quality, and contains data from a 20 % stratified sample of nonfederal acute care hospitals. The Agency for Healthcare Research and Quality allows access to the data with a signed data-use agreement. To adjust for yearly changes in the sampling design, we applied weights provided by the Agency for Healthcare Research and Quality. All analyses were performed using R, version 2.15.1 (R Foundation for Statistical Computing). Our institutional review board determined that this project was not human subjects research.

We identified all hospitalizations in adults 18 years of age and older from January 2000 through December 2011 during which either a primary or secondary procedure of ECMO was recorded. For case ascertainment, we used the *International Classification of Diseases*, *9th revision*, *Clinical Modification* (ICD-9-CM) code 39.65. We first aggregated all cases by month to produce a national sample of cases of ECMO over time. Cases were assigned to calendar month based on the date that the patient was admitted to the hospital. To comply with publication guidelines regarding low counts, prior to our analysis, we aggregated monthly counts to a 6 month period.

### Statistical analysis

The purpose of this analysis was to determine whether use of ECMO has increased during the study period and to determine if the rate of use has changed at any point during the study period. We used a segmented linear regression model to answer both questions. The model can be written as:

$$ y = \beta_{0} + \beta_{1} t + \beta_{2} \left[ {\left( {t - t_{0} } \right) * I_{cp} } \right] + \varepsilon , $$

where *y* is the dependent outcome variable (ECMO incidence), *t* is the time period (6 month interval), *t*_0_ is the change point, *I*_*cp*_ is an indicator variable for the change point, and $\varepsilon \sim N(0,  \sigma^{2} )$. The indicator *I*_*cp*_ assumes a value of 1 for time periods *t* at or beyond the change point *t*_0_, and assumes a value of 0 otherwise.

We used a data driven approach to investigate the existence and location of the change point. We visually identified a specific time period as the most plausible change point. We then fit a segmented linear regression model based on the selected change point. Residual diagnostics were conducted after de-trending the ECMO series to investigate whether there was any autocorrelation pattern in the residuals. We examined the autocorrelation function and the partial autocorrelation function for temporal correlation in the residuals, as failure to account for such correlation may lead to incorrect inferential conclusions.

Although the change point is visually evident in the time series plot, we wished to empirically validate the location of this point. We determined a time window to capture any potential change point. We then built a separate segmented linear regression model for each candidate change point within this window. The optimal change point was subsequently determined based on the model yielding the smallest value of Akaike information criterion, with optimal models corresponding to smaller values.

To determine shifts in demographics and characteristics, the sample was divided into two groups based on the identified change point. For binary outcomes (gender and mortality), comparisons of proportions were conducted using the Pearson Chi square test. For continuous outcomes (length of stay and age), comparisons of means were conducted using the Wilcoxon rank sum test.

## Results

**Fig. 1:**
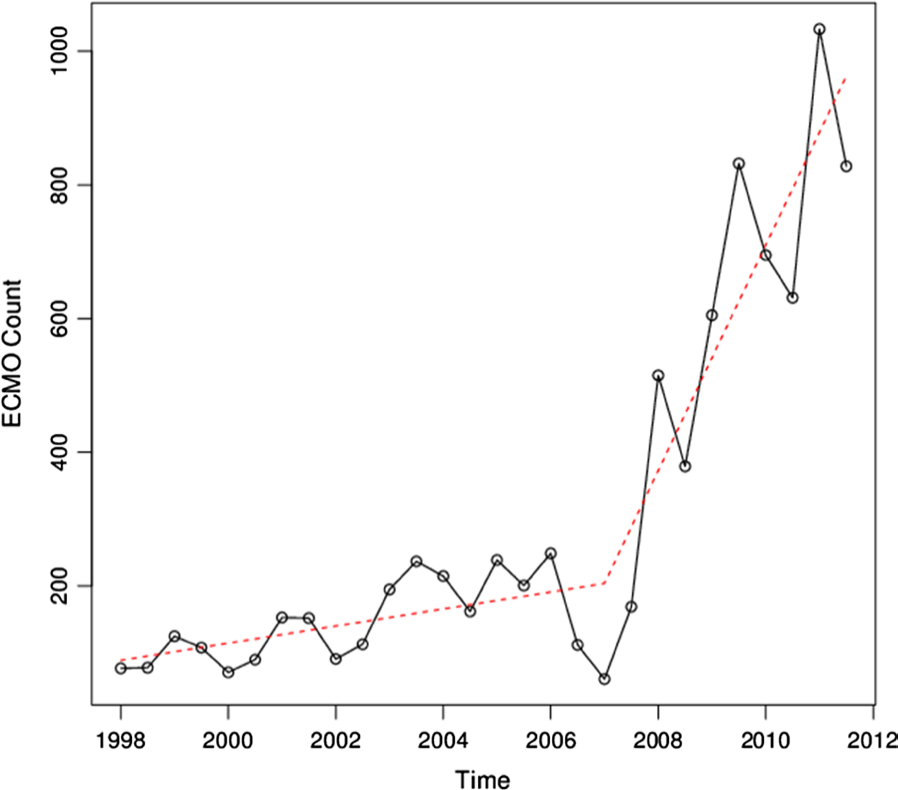
Semiannual incidence rates of hospitalizations of patients receiving extracorporeal membrane oxygenation in the United States 2000–2011 (Nationwide Inpatient Sample). The form of the fitted model is illustrated by the *dashed line segments*

Figure 1 shows a plot of overall ECMO incidence from 2000–2011. Based on a visual inspection of the plot, we chose the first half of 2007 to be the initial change point for the investigation of the autocorrelation pattern in the residuals. Upon inspection of the autocorrelation function and partial autocorrelation function, we found no evidence of temporal correlation, implying that a segmented linear regression model based on independent errors is sufficient to model the incidence of ECMO use. To confirm the time of our selected change point, we determined the time window for the coverage of the change point to be between 2005 and 2008. Based on Akaike information criterion values, the change point was confirmed to be the first half of 2007.

Our final segmented linear regression model, based on the confirmed change point, shows a significant change of slope after the first half of 2007. The form of the fitted model is illustrated in Fig. 1. The slope coefficient for the period before the change point (2007) is positive, but not statistically significant (p = 0.2595). However, a significant change of slope is detected after the change point (p = 0.0003). The large escalation in slope indicates that there is a significant effect resulting from increased use of ECMO after 2007.

Finally, we compared the demographics and characteristics. We found that for the two groups separated by the change point, there are no statistically significant differences in the mortality rate (66.2 % before 2007 vs. 63.7 % after 2007, p = 0.34) and mean age (50.5 vs. 50.4 years, p = 0.88). A higher percentage of males were treated with ECMO after the change point (56.9 % before 2007 vs. 64.3 % after 2007, p = 0.0050). Patients also had a longer mean length of stay after the change point (19.9 vs. 22.6 days, p < 0.0001).

## Discussion

Our results show that national use of ECMO in adults was stable until the first half of 2007 when there was a marked increase in frequency of use. Comparing patients who received ECMO prior to 2007 with those after 2007, we found that age and mortality were unchanged. However, length of stay has increased since 2007 and the frequency of use in males has also increased. These results indicate that there has been increasing resources invested by institutions in the implementation and use of ECMO in hospitalized patients across the nation.

Since the greatest growth in use occurred after 2007, the additional years of data in this study allows a more complete analysis of the trend over time, explaining the difference in results from prior study [6]. The reason for this growth in use after 2007 is likely multifold, and may include technical advances, improving safety of anticoagulation, as well as more aggressive interventional care in acute coronary syndrome and/or concurrent growth and acceptance of cardiovascular bridge technologies [1, 4, 8].

Despite advances in technology and increasing experience, the overall in-hospital mortality rate for patients receiving ECMO has remained relatively stable since 2000 at over 60 %. Because ECMO is primarily administered to critically ill patients, this high mortality rate may reflect the severity of underlying disease rather than the treatment itself. The mortality rate we observe is likely driven by cardiovascular disease and perioperative rescue. Patients with non-cardiovascular indications, including respiratory failure, may have a lower mortality rate, and may reflect a willingness to perform this treatment on younger, otherwise healthy patients, requiring short term life support for an acute event [6]. Registry data and other more granular data sources are important to further study mortality by indication.

A major limitation of our study is that we use administrative data. These results are based on analysis of ICD-9-CM codes only. Due to this, we are unable to assess differences in types of ECMO (e.g. veno-venous vs veno-arterial) or the individual characteristics of the patient in which it was implemented. Similarly, because the ICD-9-CM primary diagnostic admission codes varied greatly, we were unable to make meaningful conclusions regarding the underlying reason for ECMO use and how it may be changing over time. Despite these limitations, ECMO is an expensive procedure with one ICD-9-CM code; it likely that we are correctly identifying cases of ECMO use. Most importantly, and for the objective of this study, because ECMO use is uncommon, our large sample size allows us to estimate overall trends across the nation much more reliably than studies based in single centers. Our results provide a relatively unbiased view of the nation’s use of this resource through hospitalization records, complementing current registry data. With these data, we are able to explore the use of ECMO during hospitalizations on a population level across the US

In conclusion, the use of ECMO has dramatically increased in the US since 2007. Because large clinical trials will be difficult, it is important to explore indications and outcomes using observational data. As use of ECMO spreads to more centers throughout the United States, further research regarding medical and surgical care strategies, referral protocols, cost effectiveness, and resource utilization will be needed.

## Availability of supporting data

Not applicable.

## Abbreviations

ECMO: extracorporeal membrane oxygenation
ICD-9-CM: International Classification of Diseases, 9th revision, Clinical Modification
US: United States
